# Distal femoral reconstruction following failed total knee arthroplasty is accompanied with risk for complication and reduced joint function

**DOI:** 10.1186/s12891-019-2432-4

**Published:** 2019-01-31

**Authors:** Klemens Vertesich, Stephan E. Puchner, Kevin Staats, Markus Schreiner, Christian Hipfl, Bernd Kubista, Johannes Holinka, Reinhard Windhager

**Affiliations:** Department of Orthopedics and Trauma-Surgery, Medical University of Vienna, Waehringer Guertel 18-20, 1090 Vienna, Austria

**Keywords:** Megaprosthesis, Distal femoral reconstruction, Total knee arthroplasty, Complication, Revision

## Abstract

**Background:**

Substantial bone loss following failed total knee arthroplasty (TKA) represents a major challenge in revision arthroplasty, that can require distal femoral reconstruction (DFR). In this study, we aimed to assess the clinical outcome and the complication frequencies of individuals who underwent DFR with modular megaprostheses. Additionally, we aimed to compare functional outcome measures after DFR in these sophisticated cases to an age-matched control group of total knee prostheses to quantify the potential loss of function.

**Methods:**

A retrospective chart review of 30 consecutive patients after DFR from 1997 to 2017 with a mean age of 74.38 years (± 10.1) was performed. Complications were classified according to the Henderson classification. Knee Society Score (KSS) was calculated and range of motion (ROM) was assessed.

**Results:**

Thirteen (43.3%) patients had at least one complication requiring revision surgery. Revision-free survival was 74.8% at one year, 62.5% at three and 40.9% at 10 years post-op. Soft-tissue failure complications were found in three (10.0%) patients, aseptic loosening in four (13.3%) patients, structural failure in one (3.3%) patient and infection in eight (26.6%) patients. Of those with infection, five (16.6%) experienced ongoing prosthetic joint infection and three (10.0%) developed new infection after distal femur reconstruction. Patients with DFR achieved 69.3% of KSS pain score, 23.1% KSS function score and 76.2% of ROM compared to patients with primary TKA.

**Conclusions:**

DFR after failed TKA represents a treatment procedure with high risk for complication in this particular group. Despite the prospect of rapid postoperative mobilization, reduced functionality, range of motion and mobilization have to be considered when choosing this treatment option.

## Background

Revision surgery is a major issue in arthroplasty. By the year 2030, the annual number of revision total knee arthroplasty (TKA) is expected to triple [1, 2]. Patients with consecutive and/or multiple failed TKA frequently present with high-grade bone loss [3, 4]. This often limits options to restore joint function or salvage limbs and requires the use of special prostheses [5, 6].

Modular prostheses of the distal femur were initially developed for the treatment of patients who required substantial resection of the bone due to bone or soft-tissue tumors [7]. These devices are a promising alternative that could result in restoration of joint function, rapid recovery and, in extreme cases, avoid amputation [8]. Improvement of prosthetic design and biomechanics lead to enhanced functionality, motion and mobilization [9, 10]. Further, development of fixation techniques of these devices lead to improved prosthetic survival rates and reduction of revision surgeries in the group of oncologic patients [8, 11].

Individuals that have multiple complications following revision surgery often have substantial bone loss. Those individuals with periprosthetic fractures and insufficient bone healing [4, 12–14] or complex periprosthetic joint infection with consecutive reinfection may have substantial bone damage of the distal femur [15, 16]. In such cases, conventional arthroplastic and osteosynthetic methods are not sufficient for reconstruction and resection prostheses are required to restore joint function [16].

Although these prostheses promise better outcomes (more rapid mobilization and increased joint function) [17], patients face a greater risk of complications as well as potentially reduced joint functionality [16, 18].

In this study, we assessed the complication, risk, and prostheses survival frequencies of a population that underwent distal femoral reconstruction (DFR) due to substantial bone loss. Additionally, we aimed to quantify the functionality and mobilization in these patients compared to an age-matched control group of patients, who underwent primary total knee arthroplasty.

## Methods

This is a retrospective chart review of arthroplasty and reconstruction surgeries at the Medical University of Vienna approved by its Institutional Ethical Review Board. Thirty consecutive patients who underwent reconstruction of the distal femur after failed TKA between January 1997 and December 2017 were identified. The average age of the patients at the time of DFR was 74.38 years (± 10,11). There were 28 female (93.3%) and 2 male (6.7%) patients (Table 1).

**Table 1:** Demographics of patient and control group included in this study

Demographic Variable	Distal Femoral Reconstruction	Control (Total Knee Arthroplasty)	
Included	*N* = 30	*N* = 30	
Sex	Female = 28 (93.3%)	Female = 28 (93.3%)	
	Male = 2 (6.7%)	Male = 2 (6.7%)	
Age	74.38 years (±10,11)	73.0 years (±11.51)	*p* = .595
Follow-up	54.15 months (±58.67)	63.35 months (±47.57)	*p* = .409

**Fig. 1:**
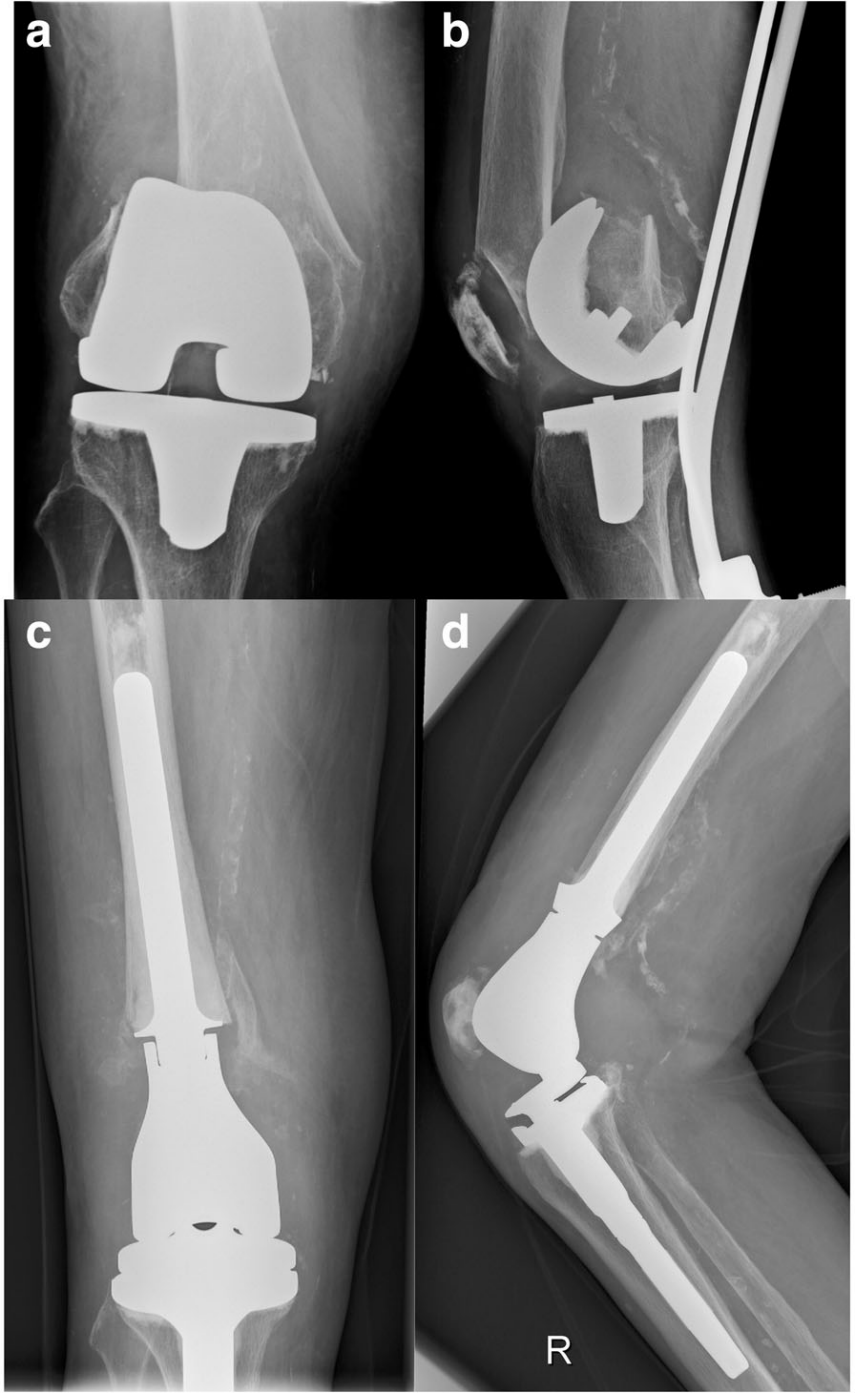
**a**, **b** Anteroposterior and lateral radiographs of an 81-year old female patient with severe periprosthetic fracture of the distal femur after trauma; **c**, **d** Anteroposterior and lateral radiograph of the same patient 4 months after consecutive reconstruction of the distal femur

**Fig. 2:**
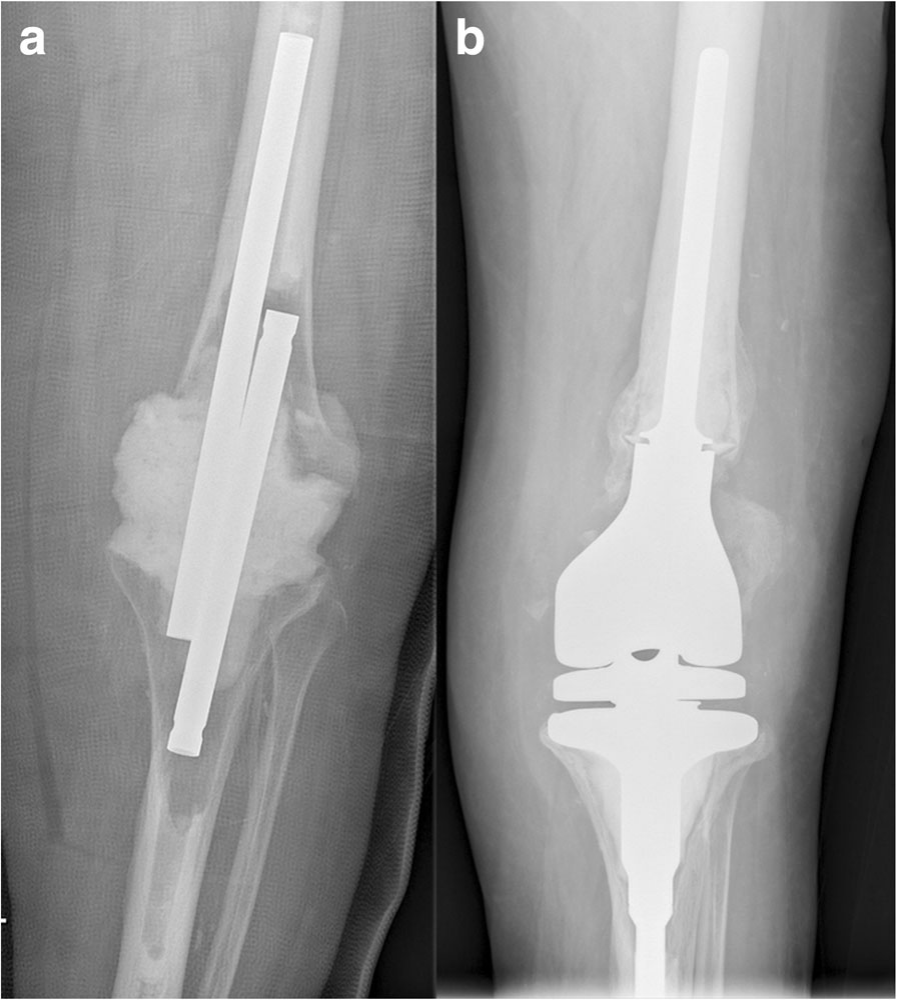
**a** Anteroposterior radiograph of a 72-year old female patient with arthrodesis and antibiotic bone cement spacer due to prosthetic joint infection after TKA; **b** Postoperative radiograph after DFR with a modular distal femoral GMRS

All DFR-procedures were performed using the Kotz Modular Femur and Tibia Reconstruction System (KMFTR, Howmedica GmbH, Kiel, Germany) in 6 (20.0%) cases, the Howmedica Modular Reconstruction System (HMRS, Howmedica GmbH, Kiel, Germany) in 3 (10.0%) cases. The Global Modular Replacement System (GMRS, Stryker Corporation, Mahwah, NJ) was used in 21 (70.0%) patients (Figs. 1 and 2).

Preoperatively radiographs in two plans as well as standing entire limb radiographs have been taken. Resection length of the femur and consecutive reconstruction was chosen to be as short as possible to provide a maximum of functionality. During the procedures, intraoperative fluoroscopy was used to control the resection length and osteotomy as well as the process of reaming for final stem implantation. Due to poor bone quality, cemented fixation of stems was used in these cases.

Follow-up examinations were performed 6 weeks, 6 months and then annually after surgery, respectively. During the follow-up clinical joint assessment, functional scoring and radiographic imaging were performed. Function and pain were recorded using the Knee Society Scoring System (KSS) and range of motion (ROM) was also assessed.

Complications of modular megaprostheses were classified as Type I (soft-tissue failure), Type II (aseptic loosening), Type III (structural failure including periprosthetic fracture and breaking of prosthetic parts), and Type IV (prosthetic joint infection), according to the Failure Mode Classification System [19]. Type V classification, progression of tumor, was not represented in the study population.

Individuals that underwent DFR after failed TKA were matched for age (± 12 months) gender and age at surgery (± 12 months) with control subjects that underwent primary TKA at our hospital (Table 1). Functional scoring and range of motion of the control group and the distal femur reconstruction group were compared to assess the difference in outcomes and the degree of potential loss of joint function in the distal femoral reconstruction group.

The mean follow-up was 54.15 ± 58.67 months (range, one to 240). The follow-up from the index or primary procedure was 136.77 ± 94.24 months and the mean time after primary TKA to DFR was 107.13 ± 83.76 months. Two patients died during the period of postoperative hospitalization on conditions not related to the disease (after 0.7 and 2 months).

Initially, all individuals in the study population underwent TKA in an attempt to alleviate the symptoms and complications of osteoarthritis.

The mean number of prior revision surgeries of the index joint was 3.17 ± 2.17 (range one to 10).

The indications for DFR were periprosthetic fracture in 13 (43.3%) cases, non-union or aseptic loosening of prosthetic or osteosynthetic devices in 8 (26.7%) cases, and periprosthetic joint infection in 9 (30.0%) cases. In these cases, patients were treated with two-stage revision and appropriate antibiotic therapy. DFR was performed if infection could be ruled out in preoperative and perioperative tests.

### Statistics

Descriptive statistics were used to describe frequencies, means and ranges of complications as well as odds ratio and relative risks in infectious patients. Revision- and complication-free survival was detected using Kaplan-Meier estimating method. Comparative analyses of functional outcome were performed by using the independent t-test for continuous variables or Mann – Whitney *U* Test for non-parametric variables, prior to being tested for normality by Kolmogorov – Smirnov test. Further, comparison of the functional scores was assessed using ANOVA within the DFR group. Additionally, regression analyses were performed to assess the correlation between the length of femoral reconstruction and outcome measurements. Statistical significance was identified as a *p*-value of less than 0.05. Statistics were performed using SPSS v24.0 (IBM Corporation, Armonk, NY, USA).

## Results

After DFR, additional revision surgery was performed on 13 (43.3%) patients after a mean period of 31.92 months (range 2 weeks to 138 months). Nine (30.0%) patients had to undergo multiple revisions.

**Fig. 3:**
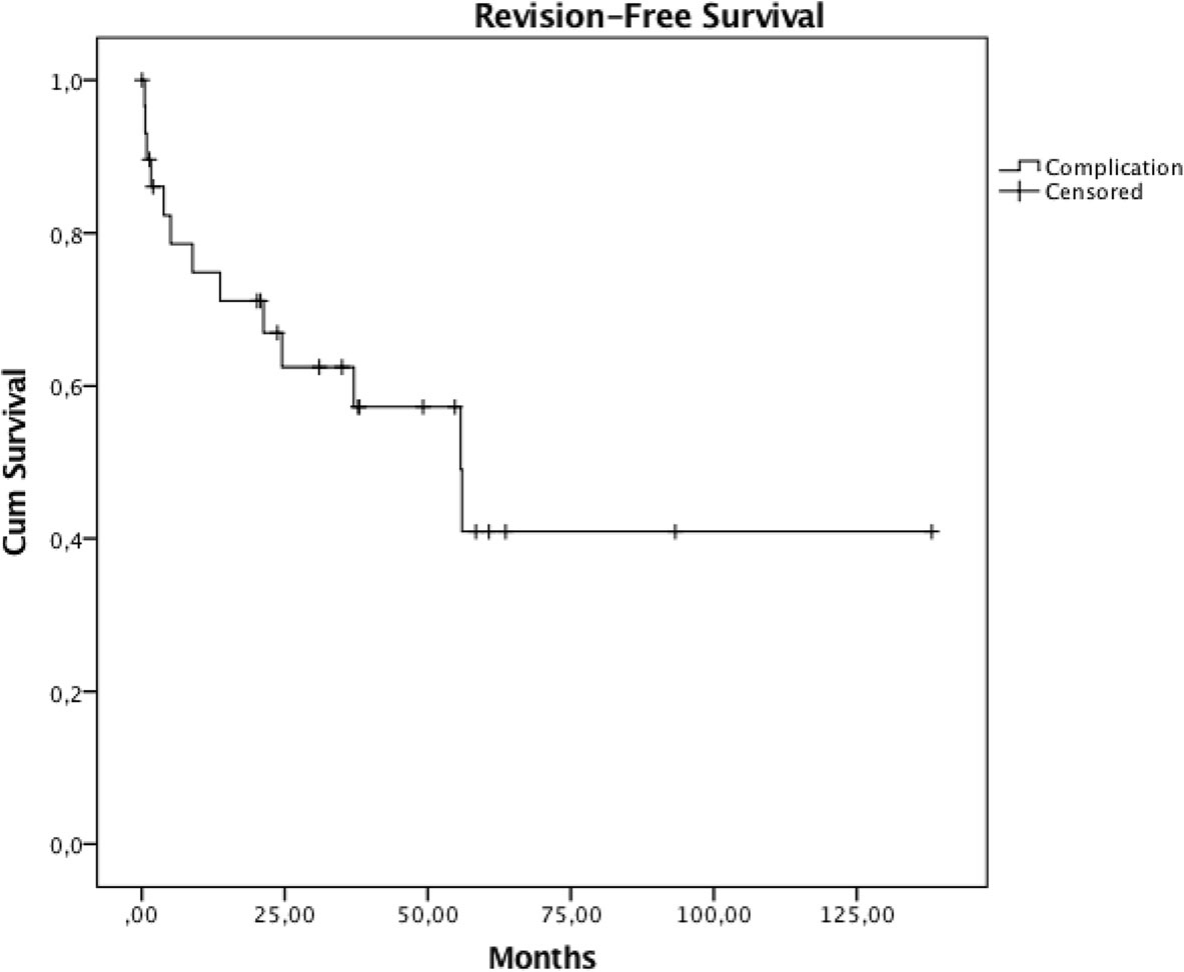
Kaplan-Meier estimation of survival of the entire distal femoral reconstruction group providing the overall survival

Overall, revision-free survival estimation was 74.8% at 1 year, 62.5% at three and 40.9% at 10 years, respectively (Fig. 3).

**Fig. 4:**
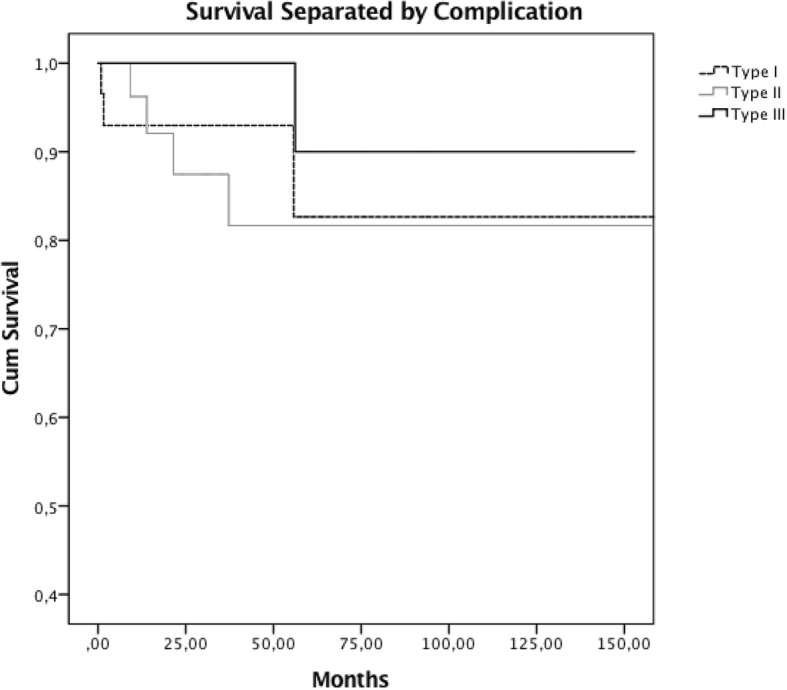
Survival using the Kaplan-Meier method separated by type of complication, Type I – Soft-tissue failure, Type II – Aseptic loosening, Type III – Structural failure

Type I complications were observed in three (10.0%) patients. These patients had recurrent patella dislocation and insufficiency or rupture of the extensor mechanism, and therefore underwent revision surgery (Fig. 4).

Four (13.3%) patients presented with Type II complications, three of which had aseptic loosening of the femoral stem and one had loosening of the tibial component. Revision surgery was sufficient in all four of these cases (Fig. 4).

One (3.3%) patient suffered from a Type III complication and underwent revision surgery due to fracture of the hinge mechanism of the prosthesis (Fig. 4).

**Fig. 5:**
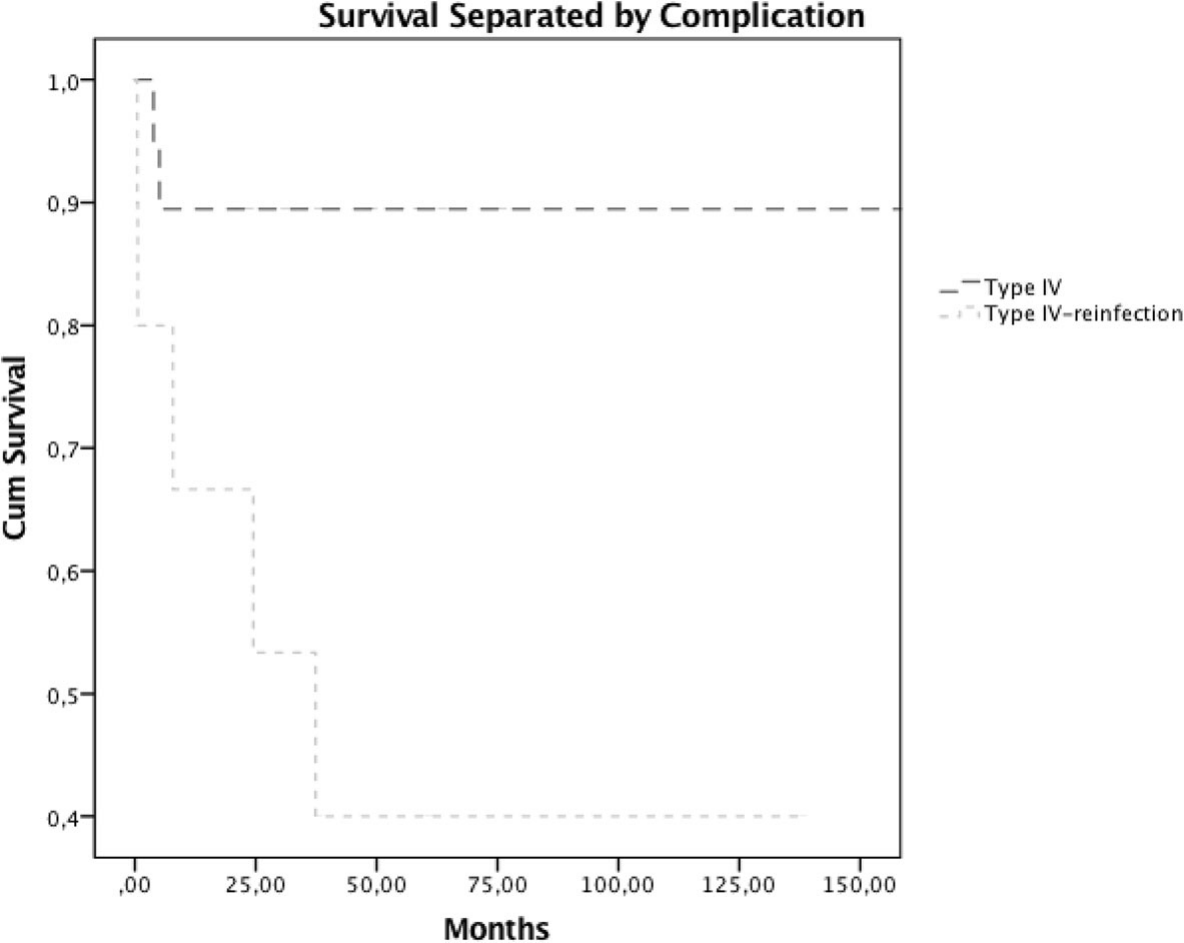
Survival estimation with Kaplan-Meier method for individuals after DFR with prior periprosthetic infection after knee prosthesis and without prior periprosthetic infection

Eight (26.6%) patients suffered from Type IV complication, five of which had recurrent infection and underwent DFR after eradication of periprosthetic joint infection of the primary prosthesis. The remaining three patients developed Type IV complication without prior infection. Based on these findings, the odds ratio was 5.667 (CI 0.990–32.429, *p* = .078). The relative risk for revision due to reinfection was 3.33 (CI 0.990–11.217, *p* = .056) if an individual previously had periprosthetic joint infection. Revision surgery and eradication of infection was sufficient in three individuals, who did not suffer from infection of the primary implant. One patient was treated with distal femoral reconstruction. Four patients had to undergo transfemoral amputation due to disease progression and additional complications. All individuals had infection of the initial joint reconstruction. The estimated revision-free survival for patients without a prior infection was 89.5% after 10 years compared to 40.0% after 10 years for patients with a previous infection, respectively (Fig. 5).

The mean post-operative KSS pain and function scores for the DFR group were 61.23 ± 21.93 (range 0–95) and 20.33 ± 19.16 (range 0–55), respectively. The mean range of motion was 82° ±16.57 (range 50–130°). Statistical analyses showed no significance in the functional outcome (ANOVA *p* > 0.05) (Table 2).

**Table 2:** Knee Society Score (KSS) and range of motion (ROM) of patients after distal femoral reconstruction separated by complication. There was no significant difference when comparing each complication type with the no complications group

	KSS pain		KSS function		ROM	
	Mean (SD)	p	Mean (SD)	p	Mean (SD)	p
No complication (*N* = 17)	63.29 (22.34)		22.06 (20.39)		85° (17.50)	
Complication (*N* = 13)	58.54 (21.98)	.914	18.08 (17.97)	.648	77° (14.80)	.616
Type I (*N* = 3)	72.00 (7.94)	.226	31.67 (7.64)	.306	88° (12.58)	.562
Type II (*N* = 4)	62.50 (10.79)	.369	16.25 (19.31)	.546	80° (8.16)	.110
Type III (N = 1)	89.00 (−)	.148	35.00 (−)	.516	100° (−)	.196
Type IV (*N* = 8)	54.13 (26.33)	.599	16.25 (17.88)	.661	74° (17.6)	.805

(*SD* Standard deviation)

**Fig. 6:**
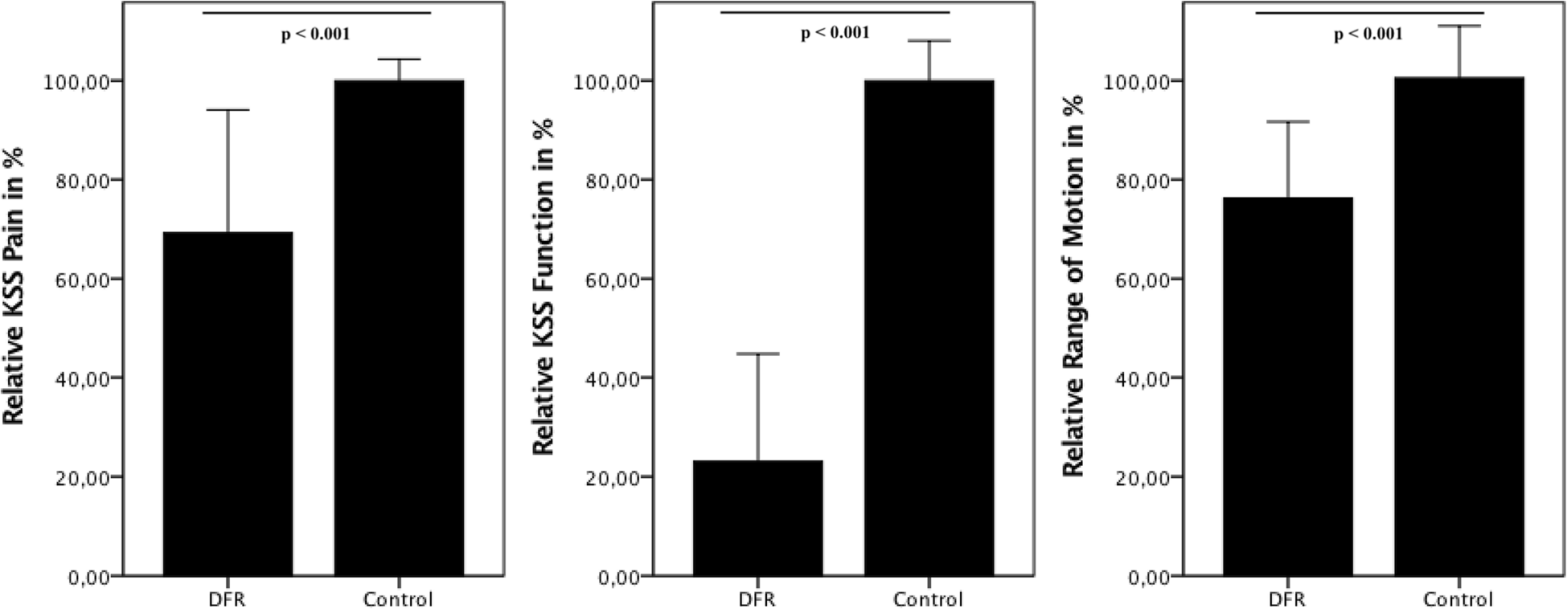
Functional results of the distal femoral reconstruction group (DFR) in comparison with the results of the control group. DFR group had significantly lower KSS scores and ROM than the control group (*p* < 0.001)

Compared to the control group (KSS pain 88.37 ± 3.78, KSS function 90.17 ± 7.25, ROM 108° ±11.27), mean values after distal femur reconstruction were significantly lower (*p* < 0.01). After distal femoral reconstruction, individuals achieved 69.3% of KSS pain score (*p* < 0.001) and 23.1% KSS function score (*p* < 0.001). Patients with DFR achieved 76.2% of ROM compared to primary TKA (*p* < 0.001) (Fig. 6).

**Fig. 7:**
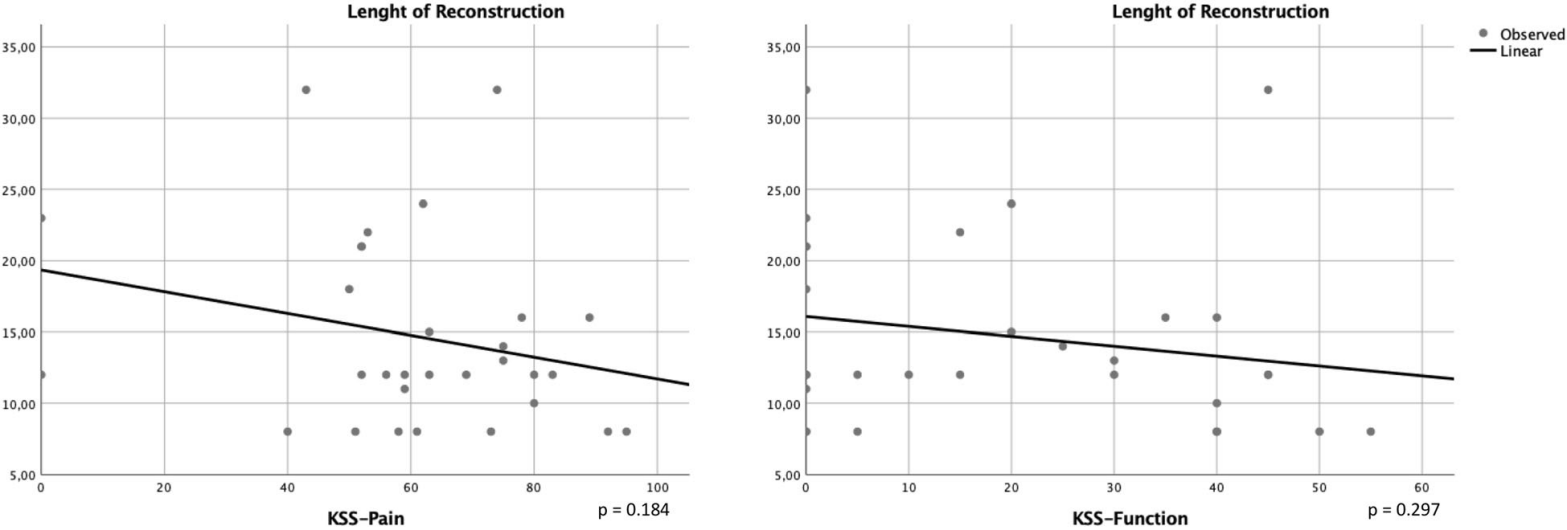
Regression analyses indicate a correlation between resection length and KSS outcome measurements but statistical significance was not shown

The mean length of femoral reconstruction was 14.67 cm (± 6.73). Regression analysis showed no significant correlation between the occurrence of any complications and the length of femoral reconstruction (*p* = 0.463). Further, regression analyses comparing the length of femoral reconstruction to KSS pain and functional scores indicated slightly inferior results for patients with increased reconstruction length yet results did not show a statistical significance (ANOVA reconstruction length and KSS pain: *p* = 0.184; ANOVA reconstruction length and KSS function *p* = 297) (Fig. 7).

## Discussion

High-grade failure following TKA represents a defiant condition of which both orthopedic surgeons and patients need to be aware. Once instability is present due to fundamental loss of bone substance, treatment options are limited [16, 20]. DFR constitutes a promising method to provide mobility, joint function and limb salvage [4]. Despite to auspicious results, literature describes distal femoral reconstruction to be associated with a distinct risk of complications and revision surgery [21].

We, therefore, aimed to assess the survival and complication frequencies after DFR in patients suffering from failed TKA. We also assessed the joint function of these patients and their function relative to a matched patient group after primary TKA.

The overall estimated survival rates of our study cohort were slightly lower, when compared to previously published studies, which observed survival rates of 80 to 40% after 10 years [9, 10, 12, 22–25]. Consistent in current literature is, however, the focus on the poor revision-free survival after DFR in non-oncological patients [22, 24]. A major issue in retrospective analyses is the limited comparability of data. There is a lack of consistency in defining revision and complication types in comparable studies, as many studies do not explicitly state which classification scheme has been used. Therefore, we decided to use a standardized classification for failure of modular megaprostheses provided by Henderson et al. to ensure the comparability of our findings [19].

The reliability of DFR represents a problem that is apparent in this study. Initially, these special prostheses were developed for patients who required substantial bone resection due to malignant diseases [8]. Compared to complication rates described in these populations, complication rates in our study population were observed to be higher [7, 11, 26]. Soft tissue and vascularity damage potentially caused by prior procedures may not be present in patients requiring DFR as primary procedure. Furthermore, bone biology in the comparably younger group of tumor patients may provide a superior environment for osseointegration, as current research describes the issue of general age-related loss of bone as a limitation for osseointegration [27–29]. The problem of advanced age and the accompanied change in bone formation may be reasons for the high rates of aseptic loosening in older individuals.

Prosthetic joint infection is a major problem with severe impact on prosthetic survival. The use of megaprostheses is associated with an increased risk for infection [6]. Generally large metal surfaces provide an environment for potential colonization of microorganisms [30–32]. Prior failed two-stage revision is also a potential risk factor for reinfection [33]. Our findings indicate that the frequency of prosthetic joint infection is higher in the group, which developed infection of the primary device and had DFR after eradication of infection. Further, the estimated survival rates describe different trends for the two separated infection groups. A poor survival of 40% in the group of individuals who had infection of the primary prostheses was assessed. Additionally, these patients had two re-revisions after DFR and required total femoral reconstruction in one case and amputation in four cases. In contrast, the infection free survival for individuals without prior infection of the artificial knee joint was 89.5% after 10 years. Generally, the management of periprosthetic joint infection is constantly evolving. Research revealed that surgical intervention with debridement and targeted antibiotic treatment is crucial for the success of intervention [34, 35]. Current recommendations for treatment suggest the two-stage exchange as algorithm of choice [36], which was utilized in the study population. Concluding on these findings, sufficient treatment of periprosthetic joint infection after DFR can be achieved in those individuals who had no infection of the primary prostheses. The risk for re-revision is elevated and the revision-free outcome after DFR is inferior for individuals who suffered already from an infected primary implant. Decision making should take prior infection into account, due to the high risk for following infections and complications and the limited treatment options and poor results after multiple reinfections [33].

Limb salvage and preservation of joint function and the prospect for rapid mobilization are the major advantages of distal femoral reconstruction for patients that have previously undergone total knee arthroplasty. This may be especially true for older patients or those with reduced physical conditions who may be mobilized sooner when the limb is preserved [17, 37]. Joint functionality after DFR in individuals with multiple revisions is expected to be at an acceptable level [9, 10, 15, 23]. Our results confirm these findings. Furthermore, the complication type did not significantly affect joint function (evaluated by KSS score) or the range of motion (Table 2). However, when comparing the DFR group with matched controls, functional results were significantly inferior in the DFR group. In most cases, mobilization was possible with crutches or with a walking frame, which is expressed as reaching just 23% of KSS functional score in the distal femoral reconstruction group.

This study has several shortcomings. Due to the retrospective character and the long observational period of two decades, surgical methods, prosthetic material and especially recommendations in the treatment of periprosthetic infection have changed over the study period. Additionally, comorbidities, that might influence the outcome of these individuals were not included due to inconsistent records. The vast majority of individuals included in the DFR group had their index procedure at external institutions and were transferred to our institution for further treatment and care. Due to that, records of joint function prior to the index procedure were not consistently accessible and not included in the analysis. Furthermore, due to the small study population, extrapolation of our results is limited. However, our sample size is similar to other studies on this particular topic [9, 10, 15, 18].

## Conclusion

Our findings show that distal femoral reconstruction after severely failed total knee arthroplasty is a procedure with high risk for complication especially when individuals have suffered from prosthetic joint infection of the primary implant. Despite the potential positive outcomes promised by this treatment, it is imperative that the possibility of serious complications (reduced functionality, decreased range of motion and limited mobilization) are considered during the decision-making process in such cases.

## Abbreviations

DFR: Distal femoral reconstruction
KSS: Knee Society Score
ROM: Range of motion
TKA: Total knee arthroplasty
